# Comparison of sporulation and germination conditions for *Clostridium perfringens* type A and G strains

**DOI:** 10.3389/fmicb.2023.1143399

**Published:** 2023-05-09

**Authors:** Marc Liggins, Norma Ramírez Ramírez, Ernesto Abel-Santos

**Affiliations:** ^1^Department of Chemistry and Biochemistry, University of Nevada, Las Vegas, Las Vegas, NV, United States; ^2^Departamento de Biología, Universidad de Guanajuato, Guanajuato, Mexico

**Keywords:** *Clostridium. perfringens*, sporulation, necrotic enteritis, spores, germination

## Abstract

*Clostridium perfringens* is a spore forming, anaerobic, Gram-positive bacterium that causes a range of diseases in humans and animals. *C. perfringens* forms spores, structures that are derived from the vegetative cell under conditions of nutrient deprivation and that allows survival under harsh environmental conditions. To return to vegetative growth, *C. perfringens* spores must germinate when conditions are favorable. Previous work in analyzing *C. perfringens* spore germination has produced strain-specific results. Hence, we analyzed the requirements for spore formation and germination in seven different *C. perfringens* strains. Our data showed that *C. perfringens* sporulation conditions are strain-specific, but germination responses are homogenous in all strains tested. *C. perfringens* spores can germinate using two distinct pathways. The first germination pathway (the amino acid-only pathway or AA) requires L-alanine, L-phenylalanine, and sodium ions (Na^+^) as co-germinants. L-arginine is not a required germinant but potentiates germination. The AA pathway is inhibited by aromatic amino acids and potassium ions (K^+^). Bicarbonate (HCO_3_^−^), on the other hand, bypasses potassium-mediated inhibition of *C. perfringens* spore germination through the AA pathway. The second germination pathway (the bile salt / amino acid pathway or BA) is more promiscuous and is activated by several bile salts and amino acids. In contrast to the AA pathway, the BA pathway is insensitive to Na^+^, although it can be activated by either K^+^ or HCO_3_^−^. We hypothesize that some *C. perfringens* strains may have evolved these two distinct germination pathways to ensure spore response to different host environments.

## Background

*Clostridium perfringens* is a Gram-positive, rod-shaped, spore-forming, anaerobic bacterium (Petit et al., 1999; Stevens et al., 2012). *C. perfringens* is a very versatile pathogen that has adapted to a number of different environments and hosts. This ability allows *C. perfringens* to causes a wide range of diseases in humans and animals (Rood and Cole, 1991).

The ability of *C. perfringens* to cause disease in different hosts is ascribed mainly to the differential production of an arsenal of protein toxins. The most important toxins linked to infections are the alpha toxin (CPA), beta toxin (CPB), enterotoxin (CPE), epsilon toxin (ETX), iota toxin (ITX), and necrotic enteritis toxin B-like (NetB) (Rood, 1998).

*Clostridium perfringens* are classified into 7 toxinotypes based on their ability to produce different toxins and infect different hosts (Rood et al., 2018). *C. perfringens* type A produces CPA as the main determinant for myonecrosis (gas gangrene). *C. perfringens* type B targets mostly sheep while producing CPACPB, and ETX. *C. perfringens* type C causes necrotic enteritis in neonatal hosts and produces both CPA and CPB, and in some strains, CPE. *C. perfringens* type D causes enterotoxaemia in *Caprinae* species using CPA and ETX. *C. perfringens* type E is not strongly associated with mammalian infections but can produce CPE and ITX (Uzal et al., 2010, 2014). *C. perfringens* type F carries the CPA gene and produce CPE upon sporulation and causes food poisoning. Finally, *C. perfringens* type G produce CPA and NetB toxin (Rood et al., 2018). NetB seems to be required for necrotic enteritis in chickens. Hence, strains obtained from diseased birds that were previously classified as type A have been reclassified as type G (Rood et al., 2018).

When stressed, *C. perfringens* forms resistant and dormant spores (Setlow, 2003). *C. perfringens* spores persist in the environment and serve as infective vehicles (Brown, 2000). To start an infection, *C. perfringens* spores need to germinate into toxin-producing vegetative cells (Paredes-Sabja et al., 2011).

Commonly, the first step to trigger spore germination is the detection of nutrients by a family of membrane proteins (Ger receptors) (Moir, 2006; Paredes-Sabja et al., 2011). Most Ger receptors are encoded in tricistronic operons that produce three distinct protein subunits that are all necessary to form a functional germination receptor (Ross and Abel-Santos, 2010).

The tricistronic operon encodes for the three proteins (A, B, and C) of the germination receptor. The A- and B-subunits have been proposed to be integral membrane proteins. The C-subunit has been proposed to be relatively hydrophilic with an N-terminal signal peptide. Recent work has implicated the B-subunit as the sensor for germinant molecules (Artzi et al., 2021; Blinker et al., 2021), while the A-subunit serves to transduce the germination signal (Amon et al., 2022).

Most *Bacilli* and *Clostridia* species encode multiple Ger receptors that allows for the recognition of different germination signals. In contrast, *C. perfringens* encodes a single tripartite functional receptor (GerK) even though the spores must be able to germinate in very different environments to cause each tissue-specific infection (Ross and Abel-Santos, 2010). As befitted for such a versatile pathogen, *C. perfringens* spores have been reported to recognize different sets of germinants. Parades-Sabja et al. reported that all *C. perfringens* spores tested use KCl and L-asparagine as a universal germination mixture (Paredes-Sabja et al., 2008). Additionally, *C. perfringens* strains carrying a chromosomal enterotoxin (*cpe*) gene can also germinate with KCl alone. In contrast, *C. perfringens* strains carrying a plasmid-borne *cpe* gene can germinate with L-alanine or L-valine, in addition to KCl/L-asparagine (Paredes-Sabja et al., 2008).

Other studies have expanded the potential germinant diversity used by *C. perfringens* spores. Wax and Frees reported that *C. perfringens* spores were also able to germinate using L-asparagine, D-glucose, D-fructose, and potassium ions (AGFK), a mixture that is commonly used to induce *B. subtilis* spore germination (Wax and Freese, 1968). A separate study found that sodium ions and phosphate instead of KCl acted as germinants of *C. perfringens* spores, but only in food poisoning isolates (Paredes-Sabja et al., 2009). In contrast, Kato et al. (2009) showed that bicarbonate, L-alanine, and inosine were capable of inducing *C. perfringens* spore germination, a response that closely mimics the germination profile of *Bacillus cereus* and *Bacillus anthracis* spores.

Differences in *C. perfringens* spore germination could be potentially attributed to dissimilar assay conditions. Indeed, previous works in *Bacillus* species have shown that sporulation environments can subsequently affect spore germination (Levinson and Hyatt, 1964; Hornstra et al., 2006; Ramirez-Peralta et al., 2011). Another possibility is that spores from different *C. perfringens* strains could recognize unique sets of germinants. Indeed, inter-strain variability in germination response has been shown in *B. cereus* spores (Hornstra et al., 2006; Dodatko et al., 2010). The heterogeneity of spore germination can be important for *C. perfringens* since each pathogenic strain may infect a limited number of hosts (Songer, 2010).

In this study, we systematically tested sporulation conditions, strain-specificity, and germinant mixtures as variables of *C. perfringens* spore germination. Our results show that spores from all *C. perfringens* strains tested germinated using two distinct pathways. In the first pathway (the amino acid-only or AA germination pathway), *C. perfringens* spores need L-phenylalanine and L-alanine as germinants. The second germination pathway (the bile salt/amino acid or BA germination pathway) is activated by mixtures of bile salts and amino acids.

## Methods

### Materials

All chemicals were purchased from Sigma-Aldrich Corporation (St. Louis, MO). Thioglycolate medium, peptones, yeast extract, raffinose and agar were purchased from VWR (West Chester, PA). *C. perfringens* strains JGS1936, JGS1473, JGS1882, JGS1521, JGS4104, JGS4151, and JGS4064 (Barbara et al., 2008) were a generous gift from Prof. J. Glenn Songer (Iowa State University, Ames, IA). These strains were selected since they were obtained from a wide variety of mammalian and avian hosts. The identities of selected *C. perfringens* spore preparations were confirmed by litmus milk test (Erickson and Deibel, 1978) and sequencing of the complete 16S rRNA gene.

### Testing of growth conditions on *Clostridium perfringens* sporulation yields

*Clostridium perfringens* strains were plated on 2% agar supplemented with 1% yeast extract, 0.1% sodium thioglycolate, 1.5% protease peptone, and 60 mM Na_2_HPO_4_. Plates were incubated overnight in an anaerobic environment (5% CO_2_, 5% H_2_, 90% N_2_). Single-cell clones were picked and grown for 4 h in either thioglycolate medium or BHI broth. All *C. perfringens* strains were then plated on 2% agar supplemented with 1% yeast extract, 0.1% sodium thioglycolate, 60 mM Na_2_HPO_4_ and 1.5% of a peptone source (protease peptone #1, protease peptone #2, protease peptone #3, or potato peptone). Media were also supplemented with 0.5% of a filter-sterilized carbon source (glucose, starch, or raffinose). Some plates were supplemented with theobromine to 0.01% final concentration. Plates were incubated for up to 14 days at 37°C under anaerobic conditions. Sporulation was quantified by microscopy observation of culture samples stained using the Schaeffer-Fulton method (Hamouda et al., 2002). Under these conditions, spores are stained green and vegetative cells are stained red. The approximate number of green free spores and red vegetative cells were counted in at least three independent microscopy fields selected at random. High level of sporulation was defined as >40% spores. Medium level of sporulation was defined as 20–40% spores. The low level of sporulation was defined as <20% spores.

### Purification of *Clostridium perfringens* spores

Each *C. perfringens* strain was plated under their best sporulation conditions (Table 1). Plates were incubated for 5–10 days at 37°C in an anaerobic environment. The resulting bacterial lawns were collected by flooding with ice-cold deionized water. Spores were pelleted by centrifugation and resuspended in fresh deionized water. After two washing steps, spores were separated from vegetative and partially sporulated cells by centrifugation through a 20–50% HistoDenz gradient. Spore pellets were washed five times with water, resuspended in 0.1% sodium thioglycolate and stored at 4°C. All spore preparations were more than 95% pure as determined by microscopy observation of Schaeffer-Fulton-stained aliquots.

**Table 1:** Source and optimal sporulation conditions for each *C. perfringens* strain.

Strain	Toxinotype	Source	Inoculum	Peptone	Theobromine
1936	A	Bovine neonatal enteritis	BHI	#1	No effect on sporulation
1882	A	Porcine necrotic enteritis	BHI	#2	Increases sporulation
1473	A	Chicken normal flora	Thioglycolate	#1	Reduces sporulation
			BHI	#3	Increases sporulation
1521	G	Chicken necrotic enteritis	Thioglycolate	#1	Required for sporulation
4064	G	Chicken necrotic enteritis	BHI	#1	Required for sporulation
4104	G	Turkey necrotic enteritis	BHI	#3	Required for sporulation
			Thioglycolate	#3	Required for sporulation
4151	A	Human gas gangrene	Thioglycolate	#2	Required for sporulation

### Preparation of germinant solutions

AGFK mixture (10 mM L-asparagine, 10 mM D-glucose, 10 mM D-fructose, 50 mM KCl) was prepared as previously described (Wax and Freese, 1968). The defined medium employed was described previously (Ramirez and Abel-Santos, 2010). Briefly, a buffer solution was made with 6.6 mM KH_2_PO_4_, 15 mM NaCl, 59.5 mM NaHCO_3_ and 35.2 mM Na_2_HPO_4_. Three solutions were prepared in using this buffer as diluent. The first solution contained all salts at 1000X concentrations (final concentration were 10 mg/L MgSO_4_•7H_2_O, 5 mg/L FeSO_4_ •7H_2_O, 5 mg/L MnCl_2_•4H_2_O). The second solution contained vitamins at 10X concentrations (final concentrations were 0.05 mg/L D-biotin, 0.1 mg/L *p*-amino benzoic acid, 0.05 mg/L thiamine hydrochloride, 0.05 mg/L pyridoxine, 1.0 mg/L nicotinic acid). The third solution contained all proteinogenic amino acids, except cysteine at 10X (final concentrations were 10 mM for each amino acid). Cysteine was prepared separately as a 10X solution in 0.2 N HCl. To prepare the defined medium, different solutions were added to buffer at the final concentrations indicated. In some samples, inosine was added to 1 mM final concentration.

To determine individual germinants, stock (10X) solutions of L-amino acids, NaHCO_3_, KHCO_3_, KCl, KBr, NaCl, NaBr, and bile salts were individually prepared in deionized sterile water. Combinations of these solutions were tested to determine germinants necessary for *C. perfringens* spore germination.

### Requirements for *Clostridium perfringens* spore germination

Changes in light diffraction during spore germination were monitored at 580 nm (OD_580_) on a Tecan Infinite M200 96-well plate reader (Tecan group, Männedorf, Switzerland). All *C. perfringens* spores were diluted to an OD_580_ of 5 and heat-activated at 65°C for 30 min (Desrosier and Heiligman, 1956). The spore suspension was cooled to room temperature and monitored for auto-germination for 30 min. Germination experiments were carried out with spores that did not auto-germinate. After heat activation, spores were resuspended to an OD_580_ of 1 in AGFK, LB broth, or defined medium. Spore germination rates were evaluated based on the decrease in OD_580_ at room temperature. After germinant additions, OD_580_ was measured at 1 min intervals for 90 min. Relative OD_580_ values were derived by dividing each OD_580_ reading by the initial OD_580_. Experiments were performed in triplicate with at least two different spore preparations (*n* ≥ 6). Germination rates were calculated from the initial linear region of the germination curves. Standard deviations were calculated from at least six independent measurements and were typically below 20%. Germination was confirmed in selected samples by microscopy observation of Schaeffer-Fulton stained aliquots.

To determine amino acid co-germinants, *C. perfringens* spores were resuspended in germination buffer (0.1 mM sodium phosphate buffer (pH 6.5), 50 mM NaHCO_3_) to an OD_580_ of 1. Putative germinants were added individually or in combinations to a final concentration of 10 mM. After addition of germinants, spore germination was monitored by the decrease in optical density at 580 nm, as above. Germination rates were set to 100% for *C. perfringens* spores germinated in the presence of L-alanine and L-phenylalanine. Relative germination for other germinant combinations was calculated as the fraction of germination rate compared to germination with L-alanine/L-phenylalanine.

To determine bile salt co-germinants, *C. perfringens* spores were resuspended in potassium phosphate buffer (pH 6.5) supplemented with 5% KHCO_3_, and 150 mM KCl. Spore germination was started by addition of 6 mM taurocholate, and 6 mM individual amino acids. *C. perfringens* spores were also germinated with 6 mM L-alanine and 6 mM individual bile salts. After addition of germinants, spore germination was monitored as above. Germination rates were set to 100% for *C. perfringens* spores germinated in the presence of L-alanine and taurocholate. Relative germination for other germinant combinations was calculated as the fraction of germination rate compared to germination with L-alanine/taurocholate.

### Testing for inhibitors of *Clostridium perfringens* spore germination

*Clostridium perfringens* spores were resuspended in sodium phosphate buffer (pH 6.5) supplemented with 5% NaHCO_3_, and 150 mM NaCl (for the AA pathway) or potassium phosphate buffer (pH 6.5) supplemented with 5% KHCO_3_, and 150 mM KCl (for the BA pathway). Spores samples were then individually supplemented with 6 mM amino acid or 6 mM bile salt analogs. Spore suspensions were incubated for 15 min at room temperature while the OD_580_ was monitored. If no germination was detected, spores were supplemented with 6 mM L-alanine/6 mM L-phenylalanine (for the AA pathway) or 6 mM L-alanine/6 mM taurocholate (for the BA pathway). Germination rates were set to 100% for *C. perfringens* spores germinated in the absence of inhibitor. Relative germination for conditions was calculated as the fraction of germination rate compared to no inhibitor.

### Effect of buffer and pH on *Clostridium perfringens* spore germination

Individual *C. perfringens* spore aliquots were individually resuspended in 0.1 M sodium phosphate buffer or 0.1 M potassium phosphate buffer and pH values were individually adjusted between 5.5 and 8.0. Germination was started by addition of 6 mM L-alanine/6 mM L-phenylalanine (for the AA pathway) or 6 mM L-alanine/6 mM taurocholate (for the BA pathway). Spore germination was monitored as above. For the AA pathway, the germination rate was set to 100% for *C. perfringens* spores germinated at pH 6.5 in sodium phosphate buffer. For the BA pathway, the germination rate was set to 100% for *C. perfringens* spores germinated at pH 6.5 in potassium phosphate buffer. The percentage of germination for other conditions was calculated as a fraction of the rate of germination at pH 6.5.

### Effect of cations and anions on *Clostridium perfringens* spore germination

*Clostridium perfringens* spores were individually incubated for 5 min in 0.1 M sodium phosphate buffer, pH 6.5 or 0.1 M potassium phosphate buffer, pH 6.5. Samples were then individually supplemented with 150 mM KCl, KBr, NaCl, NaBr, KHCO_3_ or NaHCO_3_. Germination was started by addition of 6 mM L-alanine/6 mM L-phenylalanine (for the AA pathway) or 6 mM L-alanine/6 mM taurocholate (for the BA pathway). Spore germination was monitored by the decrease in optical density, as above. For the AA pathway, the germination rate was set to 100% for *C. perfringens* spores germinated in sodium phosphate buffer without added salts. For the BA pathway, the germination rate was set to 100% for *C. perfringens* spores germinated in potassium phosphate buffer without added salts. The percentage of germination for other conditions was calculated as a fraction of the rate in the absence of added salts.

### Statistical analyses

Differences in relative germination were determined by using analysis of variance (ANOVA) followed by a Tukey–Kramer procedure (SigmaPlot v.9).

## Results

*Clostridium perfringens* have shown a lot of variability in both sporulation (Sacks, 1983; de Jong et al., 2002; Hsieh and Labbe, 2007) and germination responses (Paredes-Sabja et al., 2008; Kato et al., 2009; Paredes-Sabja et al., 2009). To determine whether these sporulation and germination differences correlated to strain origin, seven different *C. perfringens* strains were tested (Table 1). These strains were obtained from healthy and diseased avian and mammalian animals (Barbara et al., 2008; Cooper et al., 2010).

### Sporulation of *Clostridium perfringens* strains

Previous works have shown that sporulation conditions can affect the subsequent germination response of *B. cereus* and *Bacillus megaterium* spores (Hornstra et al., 2006; Ramirez-Peralta et al., 2011). To test if *C. perfringens* spore germination could be modulated by sporulation media, we created a matrix of 48 conditions for sporulation with combinations of different liquid media, solid media, carbon sources, peptones, and additives for every *C. perfringens* strain used in this study (Table 2).

**Table 2:** Conditions for *C. perfringens* sporulation.

	Replated from BHI						Replated from thioglycolate					
	Peptone number						Peptone number					
Strain	#1	#2	#3	#1*	#2*	#3*	#1	#2	#3	#1*	#2*	#3*
1936	+++	−	−	+++	−	−	−	−	−	−	−	−
1882	−	++	−	−	+++	−	−	−	−	−	−	−
4064	−	−	−	+++	−	−	−	−	−	−	−	−
1521	−	−	−	−	−	−	−	−	−	+++	−	−
4151	−	−	−	−	−	−	−	−	−	−	+++	−
1473	−	−	+++	−	−	+	++	−	−	+++	−	−
4104	−	−	−	−	−	+++	−	−	−	−	+	+++

All *C. perfringens* strains were plated on 2% agar supplemented with 1% yeast extract, 0.1% sodium thioglycolate, 60 mM Na_2_HPO_4_, 0.5% raffinose and 1.5% of a peptone source. *Plates were also supplemented with theobromine to 0.01% final concentration.

+++ >40% spores. ++ 20–40% spores. + <20% spores. − no detectable spores.

All *C. perfringens* strains tested sporulated in solid media, but not in liquid thioglycolate medium or BHI broth. However, it was observed that sporulation was dependent upon which liquid media was used for overnight growth prior to plating in agar. For strains JGS1936, JGS1882, JGS4064, overnight growth in BHI was necessary to induce sporulation upon replating in their preferred solid media. Other strains (JGS1521, JGS4151) required overnight growth in liquid thioglycolate medium to induce sporulation in agar. For other strains (JGS1473, JGS4104), the liquid media used (BHI or thioglycolate) for overnight growth changed the preference of solid media required for sporulation (Table 2).

Glucose, starch, and raffinose were tested as carbon sources for sporulation in solid media. Consistent with prior results (de Jong et al., 2002), raffinose was the preferred carbon source for *C. perfringens* sporulation (Table 2). In our hands, glucose and starch induced poor sporulation under all conditions tested (Sacks, 1983) (data not shown).

Peptone sources have been previously shown to affect the level of sporulation in *C. perfringens* strains (Hsieh and Labbe, 2007). In solid media, peptone protease #1 induced sporulation in strains JGS1936, JGS1473, JGS4064, and JGS1521. Peptone protease #2 was able to induce sporulation in strains JGS1882 and JGS4151. Peptone protease #3 induced sporulation in JGS1473 and JGS4104 (Table 2). Potato peptone can induce high levels of sporulation in some *C. perfringens* strains (Hsieh and Labbe, 2007). In our hands, however, potato peptone did not induce sporulation in any of the strains tested.

Theobromine has been reported to increase the levels of sporulation in *C. perfringens* strains (de Jong et al., 2002). Indeed, strains JGS4104, JGS4064, only sporulated robustly when theobromine was added to solid media. Meanwhile strains JGS1521, and JGS4151 sporulated strongly in plates supplemented with theobromine but only when grown in BHI broth Strains JGS1936, JGS1882, and JGS1473 sporulated in the absence of theobromine. In the presence of theobromine, sporulation levels for strain JGS1936 remained unchanged, increased for strain JGS1882, and decreased for strain JGS1473 when replated from BHI. Sporulation was unchanged for strains JGS1936 and JGS1882 and increased for strain JGS1473 with theobromine when replated from thioglycolate (Table 2).

Aside from differentially affecting sporulation levels, theobromine also reduced sporulation times in strains JGS1936, JGS1473, and JGS1882. In the absence of theobromine, sporulation was not detected until 5 days post-plating and maximum sporulation level was achieved 7–14 days post-plating. In the presence of theobromine, spores could be detected 2 days after plating and maximum sporulation levels were seen 5–7 days post-plating for all three strains (data not shown).

### Identification of amino acids and sterane germinants of *Clostridium perfringens* spores

**Figure 1:**
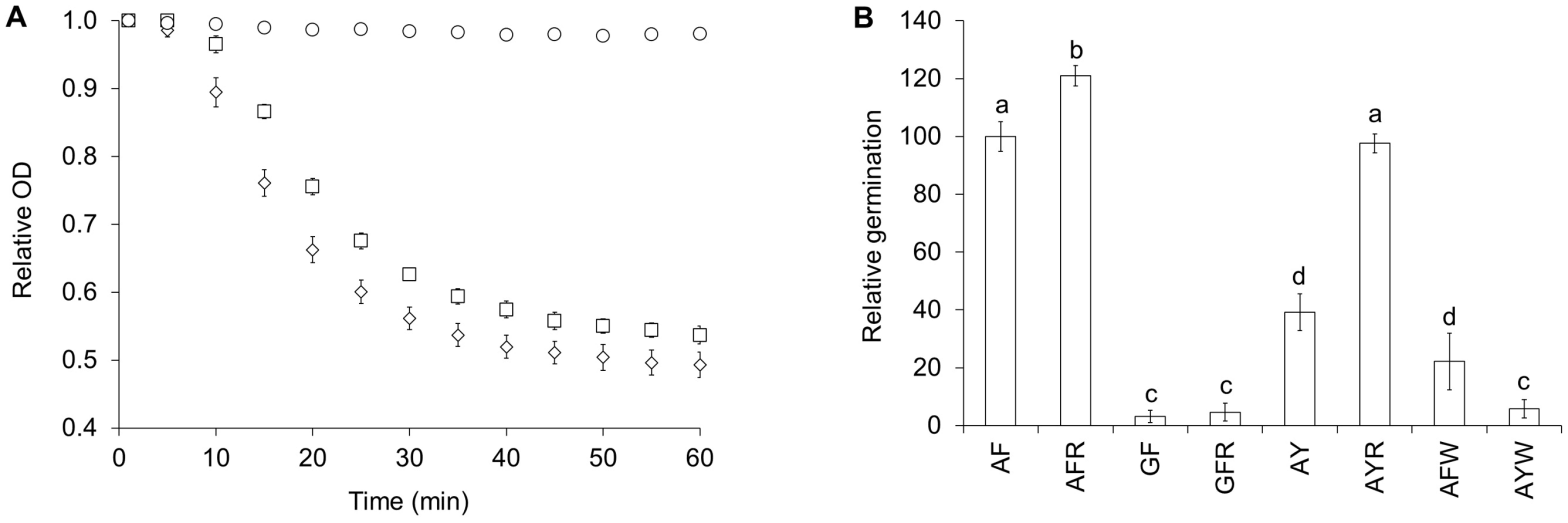
L-alanine/L-phenylalanine-mediated *C. perfringens* spore germination is potentiated by L-arginine and inhibited by L-tryptophan. **(A)** *C. perfringens* JGS1936 spores were germinated in sodium phosphate buffer (pH = 6.5) (○), defined medium (◊), or in sodium phosphate buffer (pH = 6.5) containing 10 mM L-alanine, 10 mM L-phenylalanine, and 50 mM NaHCO_3_ (□). Germination was followed by the decrease of optical density at 580 nm (OD_580_). For clarity, data are shown at 5-min intervals. **(B)** *C. perfringens* JGS1936 spores were treated with mixtures containing 10 mM L-alanine (A), L-phenylalanine (F), L-arginine (R), glycine (G), L-tyrosine (Y), and/or L-tryptophan (W). Germination rates were calculated from the linear segment of optical density changes over time. Relative germination was calculated as the fraction of each germination rate to the germination rate of spores treated with 10 mM L-alanine/10 mM L-phenylalanine. Error bars represent standard deviations of six independent measurements. Columns that are labeled with different letters are statistically different (*p* < 0.01) by one-way ANOVA followed by Tukey–Kramer multiple comparison analysis.

Unlike previous works on other *C. perfringens* strains (Paredes-Sabja et al., 2008; Kato et al., 2009; Paredes-Sabja et al., 2009), spores of *C. perfringens* strains tested in this study failed to germinate with AGFK, KCl/L-asparagine, sodium/phosphate, or L-alanine/inosine mixtures. Like other *Clostridium* species, all *C. perfringens* spores tested germinated efficiently in defined medium. Since *C. perfringens* strain JGS1936 sporulated efficiently and showed the same germination profile as all other strains, we used strain JGS1936 as model strain to analyze germination conditions for *C. perfringens* type A and type G strains (Figure 1A). *C. perfringens* spores from all strains germinated at the same rate in medium containing exclusively a mixture of all 20 proteinogenic amino acids (data not shown). Henceforth, we refer to this germination response as the amino acid-only (AA) germination pathway.

To identify which amino acids are required for germination, *C. perfringens* strain JGS1936 spores were exposed to mixtures of either small (L-alanine (A) and glycine (G)), polar (L-serine, L-threonine, and L-cysteine), hydrophobic (L-leucine, L-isoleucine, L-methionine, and L-valine), aromatic (L-phenylalanine, L-tyrosine, and L-tryptophan), basic (L-arginine, L-lysine, and L-histidine), acidic (L-aspartic acid and L-glutamic acid), amide (L-asparagine and L-glutamine), or constrained (L-proline) amino acids. None of these solutions alone was sufficient to trigger spore germination (data not shown). *C. perfringens* spores were then resuspended in solutions containing all possible pairs and trios combinations of the above amino acid groups. *C. perfringens* spore germination was only observed in solutions containing mixtures of small and aromatic amino acids. Faster *C. perfringens* spore germination rates were observed when small and aromatic amino acids were supplemented with basic amino acids (Supplementary Table S1).

To further narrow the identity of L-amino acid germinants, all 18 possible combinations of small, aromatic, and basic amino acids were tested individually for their effect on *C. perfringens* strain JGS1936 spore germination. Strong *C. perfringens* spore germination was seen in the presence of L-alanine/L-phenylalanine (Figure 1B). L-arginine was not required to trigger germination, but increased germination rates by 20% (Figure 1B). In the L-alanine/L-phenylalanine germination mixture, L-alanine could not be substituted for glycine, even in the presence of L-arginine. L-tyrosine can substitute L-phenylalanine, but the germination rate was more than 50% slower (Supplementary Table S1). The addition of L-arginine to L-alanine/L-tyrosine-treated spores increased germination rates more than two-fold. *C. perfringens* spores did not respond to L-alanine/L-tryptophan mixtures. In fact, L-tryptophan behaved as an inhibitor of L-alanine/L-phenylalanine-mediated *C. perfringens* spore germination (Figure 1B). Spores from all other *C. perfringens* strains tested were able to germinate using the AA pathway and their germination profiles were undistinguishable from *C. perfringens* strain JGS1936 spores (Supplementary Figure S1A).

**Figure 2:**
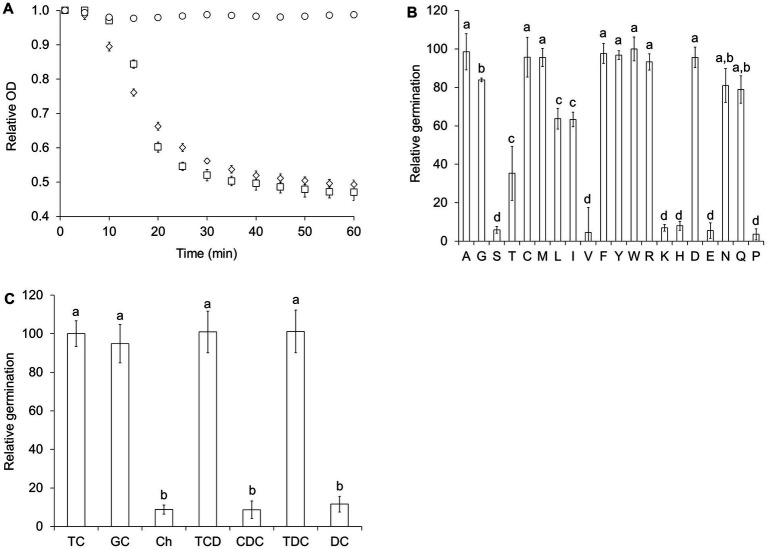
*C. perfringens* spores germinate with bile salts and amino acids. **(A)** *C. perfringens* JGS1936 spores were germinated in potassium phosphate buffer (pH = 6.5) (○), defined medium (◊), or in potassium phosphate buffer (pH = 6.5) containing 6 mM L-alanine/6 mM taurocholate (□). **(B)** *C. perfringens* JGS1936 spores were treated with 6 mM taurocholate and 6 mM of each individual amino acid. Amino acids are represented by their one-letter code. **(C)** *C. perfringens* JGS1936 spores were treated with 6 mM L-alanine supplemented with either 6 mM taurocholate (TC), glycocholate (GC), cholate (Ch), taurochenodeoxycholate (TCD), chenodeoxycholate (CDC), taurodeoxycholate (TCD), or deoxycholate (DC). Relative germination was calculated as the fraction of each germination rate to the germination rate of spores treated with 6 mM L-alanine/6 mM taurocholate. Error bars represent standard deviations of six independent measurements. Columns that are labeled with different letters are statistically different (*p* < 0.01) by one-way ANOVA followed by Tukey–Kramer multiple comparison analysis.

We recently showed that sterane compounds can modulate the germination response of *Clostridioides difficile* and *Clostridium sordellii* spores (Liggins et al., 2011). Taurocholate, a known germinant of *C. difficile* spores (Sorg and Sonenshein, 2008; Howerton et al., 2011), was not sufficient to induce germination in *C. perfringens* spores (data not shown). However, mixtures of taurocholate and L-alanine induced strong germination response (Figure 2A). Furthermore, combinations of taurocholate and a variety of amino acids also induced *C. perfringens* spore germination (Supplementary Table S1). Eight L-amino acids (L-cysteine (C), L-methionine (M), L-phenylalanine (F), L-tyrosine (Y), L-tryptophan (W), L-arginine (R), and L-aspartic acid (D)) were as effective as L-alanine (A) as co-germinants of taurocholate (Figure 2B). On the other hand, glycine (G), L-asparagine (N), and L-glutamine (Q) were slightly less effective as co-germinants. In turn, L-threonine (T), L-leucine (L), and L-isoleucine (I) were still able to synergize with taurocholate to trigger *C. perfringens* spore germination, but the germination rate was slowed by approximately 50% (Supplementary Table S1). In fact, only six amino acids (L-serine (S), L-valine (V), L-lysine (K), L-histidine (H), L-glutamic acid (E), and L-proline (P)) did not synergize with taurocholate to induce significant *C. perfringens* spore germination (Figure 2B). In addition to taurocholate, glycocholate, taurochenodeoxycholate, and taurodeoxycholate also induced *C. perfringens* spore germination in the presence of the same amino acids that trigger taurocholate-mediated germination (Supplementary Table S1). Cholate, chenodeoxycholate, and deoxycholate did not induce nor inhibit *C. perfringens* spore germination in the presence of L-alanine (Figure 2C). Henceforth, we refer to this germination response as the bile salt/amino acid (BA) germination pathway.

Similar to the AA pathway, spores from all *C. perfringens* strains tested were able to germinate using the BA pathway and their germination profiles were undistinguishable from *C. perfringens* strain JGS1936 spores (Supplementary Figure S1B).

Because bile salts serve to solubilize dietary fats (Coleman, 1987), we treated *C. perfringens* spores with either SDS or Triton-X-100. Neither detergent was able to trigger *C. perfringens* spore germination even in the presence of excess L-alanine (data not shown).

D-amino acids have been shown to inhibit amino acid-mediated spore germination in *Bacillus* species (Yasuda and Tochikubo, 1984). D-alanine and D-arginine failed to inhibit or induce *C. perfringens* spore germination in the AA germination pathway, but D-phenylalanine and D-tryptophan inhibited this pathway. In contrast, all the D-amino acids tested served as co-germinants with taurocholate in the BA pathway (data not shown).

### Optimization of *Clostridium perfringens* spore germination conditions

**Figure 3:**
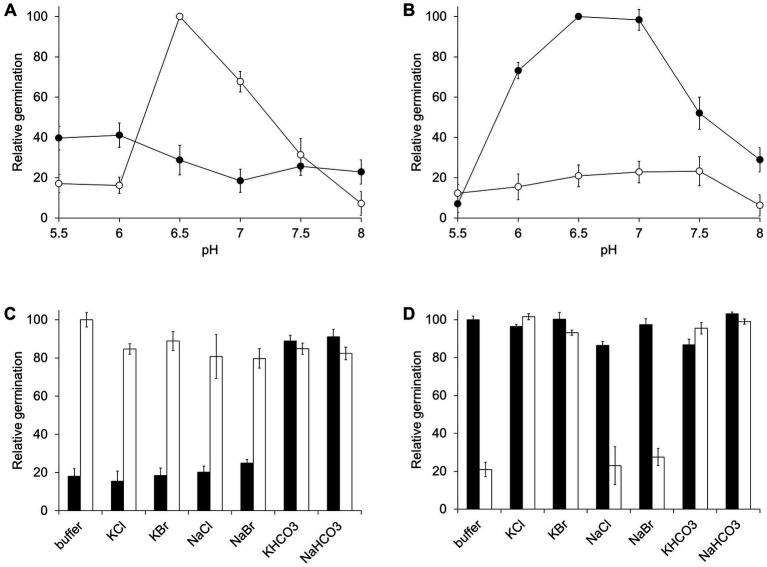
Effect of pH and ions on *C. perfringens* spore germination. **(A)** *C. perfringens* JGS1936 spores were treated with 6 mM L-alanine and 6 mM L-phenylalanine at different pH values in sodium phosphate buffer (○) or potassium phosphate buffer (●). Relative germination was calculated as the fraction of each germination rate to the germination rate for spores suspended in sodium phosphate buffer, pH = 6.5. Error bars represent standard deviations of six independent measurements. **(B)** *C. perfringens* JGS1936 spores were treated with 6 mM taurocholate and 6 mM L-alanine at different pH values in sodium phosphate buffer (○) or potassium phosphate buffer (●). Relative germination was calculated as the fraction of each germination rate to spores suspended in potassium phosphate buffer, pH = 6.5. **(C)** *C. perfringens* JGS1936 spores were resuspended in either sodium phosphate buffer (white bars) or potassium phosphate buffer (black bars). Samples were then individually supplemented with 150 mM KCl, KBr, NaCl, NaBr, KHCO_3_ or NaHCO_3_. Germination was initiated by addition of 6 mM L-alanine/6 mM L-phenylalanine. Relative germination was calculated as the fraction of each germination rate to spores suspended in non-supplemented sodium phosphate buffer, pH = 6.5. **(D)** *C. perfringens* JGS1936 spores were resuspended in either sodium phosphate buffer (white bars) or potassium phosphate buffer (black bars). Samples were then individually supplemented with 150 mM KCl, KBr, NaCl, NaBr, KHCO_3_ or NaHCO_3_. Germination was initiated by addition of 6 mM taurocholate/6 mM L-alanine. Relative germination was calculated as the fraction of each germination rate to spores suspended in non-supplemented potassium phosphate buffer, pH = 6.5.

To define the optimal conditions for *C. perfringens* germination, spores were germinated at different pH values. In the AA pathway, germination was significantly reduced if sodium phosphate buffer was substituted with potassium phosphate buffer (Figure 3A). In contrast, the BA pathway was only active in the presence of potassium ions (Figure 3B). For both pathways and in all strains, optimal germination occurred at near neutral pH. Germination was significantly reduced above pH 7.5 or below pH 5.5.

Interestingly, addition of KCl, KBr, NaCl, or NaBr did not affect the AA pathway response in either potassium phosphate or sodium phosphate buffer (Figure 3C). Similarly, NaCl and NaBr did not affect the BA germination pathway when spores were resuspended in potassium phosphate or sodium phosphate buffer. On the other hand, addition of KCl or KBr induced the BA pathway in spores resuspended in sodium phosphate buffer (Figure 3D).

Bicarbonate has been shown to be an essential co-germinant for some *Clostridium* species (Kato et al., 2009; Ramirez and Abel-Santos, 2010). In both the AA and BA germination pathways, addition of potassium bicarbonate or sodium bicarbonate increased germination rate for *C. perfringens* spores resuspended in potassium and sodium phosphate buffers, respectively (Figures 3C,D).

Because *C. perfringens* spores responded to germinants in a manner similar to *C. sordellii* and *C. difficile*, we needed to rule out the possibility of contamination with these *Clostridia* species. Hence, we tested all spore preparations by germination and growth in litmus milk medium, a test commonly used to differentiate different *Clostridia*. As expected for *C. perfringens*, all samples showed stormy clot fermentation (Erickson and Deibel, 1978). The identities of selected spore samples were further confirmed by repeating 16S rRNA sequencing.

## Discussion and conclusion

*Clostridium perfringens* is an animal pathogen with wide host distribution (Songer, 2010). Pathogenic *C. perfringens* strains seem to be host specific (Cooper et al., 2010). As proof-of-principle to test if *C. perfringens* spores could impact host selection, we investigated the sporulation and germination profiles of pathogenic and non-pathogenic *C. perfringens* strains obtained from different hosts. These strains were all originally classified as toxinotype A (Barbara et al., 2008). However, since NetB has been shown to be necessary for necrotic enteritis in fowl, strains obtained from diseased birds have been reclassified as toxinotype G (Rood et al., 2018).

*Clostridium perfringens* sporulation showed large strain variability. The source of peptone, the presence of theobromine, and the liquid media used for overnight bacterial growth contributed to this variability. Some strains sporulated under a single condition tested, while other strains had less stringent sporulation requirements. It is not clear why conditions changes and additives had such profound effects on *C. perfringens* sporulation.

There was no apparent relationship between sporulation conditions and *C. perfringens* strain pathogenicity or host source. This is not entirely surprising since sporulation might not occur inside the nutrient-rich host but when cells are in a starvation-prone environment. The almost random nature of sporulation conditions for *C. perfringens* strains is certainly intriguing and needs to be investigated further. The best sporulation conditions for each strain are shown in Table 1.

Although *C. perfringens* sporulation conditions varied greatly between strains, the germination response was homogenous. Spores from different *C. perfringens* strains prepared under similar conditions behaved identically in germination assays. Furthermore, spores from a single strain prepared under different conditions were undistinguishable in germination assays (data not shown). These results contrast with previous work suggesting that sporulation conditions affected the germination response of *Bacillus* spores (Levinson and Hyatt, 1964; Hornstra et al., 2006; Ramirez-Peralta et al., 2011).

Contrary to previous work, KCl, inosine, asparagine, glucose, fructose, sodium, and phosphate were unable to trigger *C. perfringens* spore germination (Paredes-Sabja et al., 2008; Kato et al., 2009; Paredes-Sabja et al., 2009). The differences with these studies might be due to inter-strain variations. On the other hand, we have previously shown that *Bacillus* spores purified by water washing alone show different germination profiles than spore preparations purified by gradient centrifugation (Abel-Santos and Dodatko, 2007). Similar results were observed for *C. perfringens* spores. Water-washed spores were more heterogeneous and contained larger amounts of cellular debris. These contaminants can synergize with added metabolites to produce spurious germination responses not seen in cleaner spore preparation. Indeed, *C. perfringens* spore preparations purified by water washing auto-germinate upon storage (data not shown). This heterogeneity could be more pronounced in *C. perfringens* spores due to the sensitivity of the germination response to the nature and order of addition of cations and bicarbonate.

Since all strains showed the same germination response with germination mixtures, we decided to use spores from *C. perfringens* strain JGS1936 to identify individual metabolites required to trigger germination. We selected this strain because it showed a strong sporulation profile that provided an ample spore supply.

**Figure 4:**
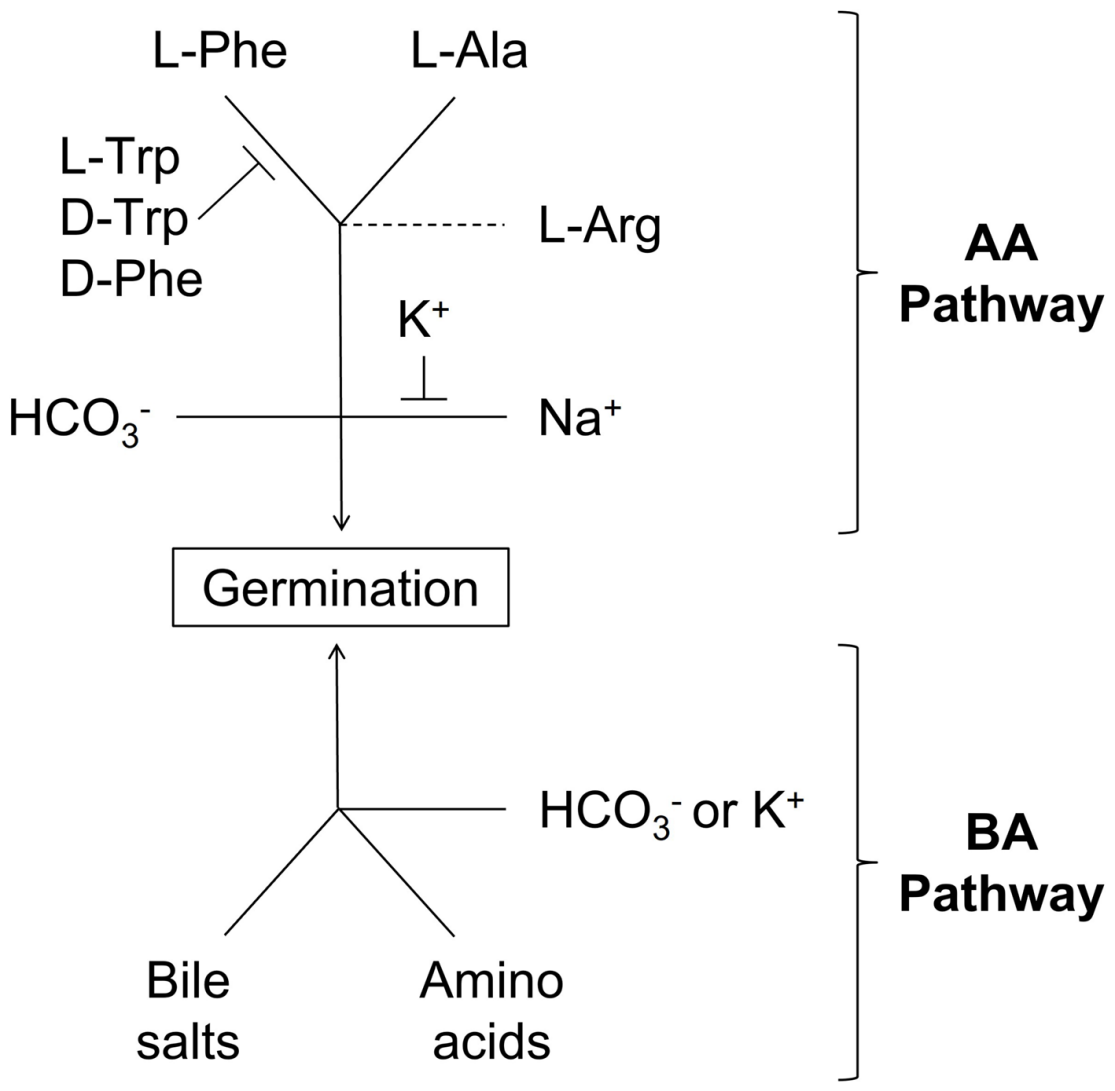
Scheme for *C. perfringens* spore germination through the AA and BA germination pathways: Solid lines represent required co-germinants. Capped lines represent germination inhibitors. Dashed lines represent germination enhancers.

Detailed analysis of germination conditions revealed two independent germination pathways (Figure 4). The AA pathway required, at a minimum, L-alanine and L-phenylalanine to induce strong *C. perfringens* spore germination. L-arginine was not required but potentiates *C. perfringens* spore germination through the AA pathway. The competitive inhibition of the AA pathway by L-tryptophan, D-tryptophan, and D-phenylalanine suggests that binding of L-phenylalanine is a key process in triggering this germination response.

The AA pathway also requires either bicarbonate or sodium ions. Sodium ions were necessary to activate the AA pathway in the absence of bicarbonate. Potassium acts as an inhibitor of the AA pathway, but only when added before sodium. We postulate that binding of cations is very fast and irreversible since once potassium is bound, sodium cannot induce germination of *C. perfringens* spores. Concomitantly, once sodium is bound, potassium is unable to inhibit germination. The presence of bicarbonate made the AA pathway insensitive to both potassium and sodium. This suggests that bicarbonate activates a separate, potassium insensitive, AA germination pathway.

In contrast to the limited number of amino acids used in the AA germination pathway, the BA germination pathway could be triggered by multiple combinations of amino acids and conjugated bile salts. Indeed, while L-tryptophan, D-tryptophan, and D-phenylalanine inhibited the AA pathway, these amino acids were strong co-germinants of the BA pathway.

The presence of salts also affected the BA germination pathway. The salt effect, however, was qualitatively different than in the AA pathway. Potassium, an inhibitor of the AA pathway, acted as activator of the BA pathway. In contrast, sodium ions did not inhibit the BA pathway. Bicarbonate was also a strong activator of the BA pathway, bypassing potassium requirement. Neither pathway is activated or inhibited by phosphate, bromide, or chloride.

Both the AA and BA pathways were discovered using spores from *C. perfringens* strain JGS1936. However, all other strains were tested for germination using the best conditions determined for both pathways (Supplementary Figures S1A,B). Since the germination profiles for all other strains were identical to *C. perfringens* strain JGS1936 spore germination, we hypothesize that the AA and BA pathways are universally conserved among *C. perfringens* type A and type G.

The *C. perfringens* spores tested share germination characteristics with both *C. sordellii* and *C. difficile*. The AA pathway was similar (but not identical) to the germination requirements of *C. sordellii* spores (Ramirez and Abel-Santos, 2010). On the other hand, the BA germination pathway was similar (but not identical) to the germination requirements of *C. difficile* spores (Ramirez et al., 2010; Howerton et al., 2011).

The similarities between bile-salt mediated germination of *C. perfringens* and *C. difficile* is intriguing. *C. difficile* spores lack Ger receptors but instead seems to use the CspABC protease complex to recognize bile salts (Francis et al., 2013; Bhattacharjee et al., 2016; Kevorkian and Shen, 2017; Rohlfing et al., 2019; Shrestha et al., 2019). *C. perfringens* also encode CspA and CspC but the role of these proteins as *C. perfringens* germination receptors has not been established.

Recognition of bile salts by *C. perfringens* suggests that the sterane backbone is an ancestral germination signal as both *C. difficile* and *C. sordellii* recognize bile salts and progesterone analogs (Liggins et al., 2011). Whereas *C. sordellii* only uses steranes as enhancers of amino-acid mediated spore germination, both *C. difficile* and *C. perfringens* use bile salts as effective co-germinants. However, *C. perfringens* recognize a broader set of sterane germinants compared to *C. difficile*. More importantly, *C. perfringens* spores, similar to *C. sordellii*, are able to germinate using only amino acids as germination signals.

*Clostridium perfringens* may have evolved multiple germination pathways to ensure spore response to different host environments. During the start of myonecrosis, *C. perfringens* spores would be exposed to abundant amino acid concentrations from decaying soft tissues. Furthermore, sodium and bicarbonate will be highly abundant in the fluids surrounding cells (Hill, 1990). These are optimal conditions to activate the AA germination pathway (Figure 4, top).

In enteric infections, however, *C. perfringens* spores would be exposed to the gastrointestinal tract environment. In the GI tract, bile salts and amino acids are abundant. Furthermore, potassium concentrations are up to 20-fold higher than in serum (Sorensen et al., 2010). These signals would favor using the BA germination pathway (Figure 4, bottom) to establish infection.

Intriguingly, only conjugated bile salts were able to trigger *C. perfringens* spore germination. Conjugated bile salts are produced in the liver and are rapidly hydrolyzed by the normal gut microbiota. Hence, conjugated bile salts accumulate at higher concentrations during conditions of dysbiosis (e.g., aggressive antibiotic treatment). Whether, similar to *C. difficile*, antibiotic treatment could mediate intestinal *C. perfringens* infections has not been established.

The unorthodox germination behavior of *C. perfringens* spores is further highlighted by the fact that all strains sequenced encode a single, intact GerK receptor (Ross and Abel-Santos, 2010). A BLAST analysis shows that the GerK protein is well conserved (≥98% identity) among *C. perfringens* strains. Thus, if strain-specific germination responses are dependent on GerK, this receptor must be able to recognize structurally different molecules. This is highlighted by the diversity of amino acid and bile salts recognized by the more promiscuous BA pathway.

Hence, the GerK receptor must have multiple binding sites to be able to recognize diverse germinants and change substrate specificity between strains. Alternatively, some of the germinants could be recognized by unidentified germination receptors, such as the CspABC complex (Shah et al., 2008). Furthermore, some *C. perfringens* strains carry an orphan B-subunit gene that has been implicated in the changing germinant preference. Whether the AA or BA pathway are dependent on Gerk and/or CspABC activation is not known and will need to be experimentally probed in the future.

## Data availability statement

The raw data supporting the conclusions of this article will be made available by the authors, upon request.

## Author contributions

EA-S directed the research, analyzed the data, and wrote the manuscript. ML and NR performed the experiments, analyzed the data, and edited the manuscript. All authors contributed to the article and approved the submitted version.

## Funding

This publication was made possible by grants from the National Institute of Food and Agriculture, U.S. Department of Agriculture, under Agreement No. 2010-65119-20603 and the National Institute of General Medical Sciences (GM103440) from the National Institutes of Health.

## Conflict of interest

The authors declare that the research was conducted in the absence of any commercial or financial relationships that could be construed as a potential conflict of interest.

## Publisher’s note

All claims expressed in this article are solely those of the authors and do not necessarily represent those of their affiliated organizations, or those of the publisher, the editors and the reviewers. Any product that may be evaluated in this article, or claim that may be made by its manufacturer, is not guaranteed or endorsed by the publisher.

## In memoriam

This article is dedicated to Prof. J. Glenn Songer (1950–2021), a pioneer in the study of *C. perfringens* diseases.
