# Supplementary material for: Comparison of sporulation and germination conditions for Clostridium perfringens type A and G strains
Source: Front Microbiol. 2023 May 9;14:1143399. doi: 10.3389/fmicb.2023.1143399 (PMC10203408; doi:10.3389/fmicb.2023.1143399)
Supplement: Supplementary file 1 [file Table_1.DOCX]

**Supplementary tables**

| **Table S1. Germination rates for the AA and BA pathways** | | |
| --- | --- | --- |
| **AA pathway** | | |
| **Condition** | **Germination rates (ΔOD/min)** | **Stdev** |
| Buffer | 4.10E-04 | 2.9E-06 |
| Defined medium | 1.96E-02 | 5.86E-04 |
| AF | 1.87E-02 | 9.64E-04 |
| AFR | 2.26E-02 | 6.74E-04 |
| GF | 5.98E-04 | 3.93E-04 |
| GFR | 8.60E-04 | 5.80E-04 |
| AY | 7.33E-03 | 1.19E-03 |
| AYR | 1.82E-02 | 6.10E-04 |
| AFW | 4.16E-03 | 1.83E-03 |
| AYW | 1.08E-03 | 5.98E-04 |
| *C. perfringens* JGS1936 spores were treated with mixtures containing L-alanine (A), L-phenylalanine (F), L-arginine (R), glycine (G), L-tyrosine (Y), and/or L-tryptophan (W). Germination rates were calculated from the linear segment of optical density changes over time | | |
| **BA pathway** | | |
| **Condition** | **Germination rates (ΔOD/min)** | **Stdev** |
| Buffer | 8.8E-05 | 2.4E-06 |
| TC + A | 1.92E-02 | 1.82E-03 |
| TC + G | 1.63E-02 | 1.77E-04 |
| TC + S | 1.14E-03 | 3.34E-04 |
| TC + T | 6.88E-03 | 2.73E-03 |
| TC + C | 1.87E-02 | 2.02E-03 |
| TC + M | 1.86E-02 | 9.18E-04 |
| TC + L | 1.24E-02 | 1.05E-03 |
| TC + I | 1.23E-02 | 7.29E-04 |
| TC + V | 8.92E-04 | 2.53E-03 |
| TC + F | 1.90E-02 | 1.01E-03 |
| TC + Y | 1.89E-02 | 4.45E-04 |
| TC + W | 1.95E-02 | 1.21E-03 |
| TC + R | 1.82E-02 | 8.27E-04 |
| TC + K | 1.36E-03 | 3.49E-04 |
| TC + H | 1.56E-03 | 4.59E-04 |
| TC + D | 1.86E-02 | 1.03E-03 |
| TC + E | 1.07E-03 | 7.89E-04 |
| TC + N | 1.58E-02 | 1.71E-03 |
| TC + Q | 1.54E-02 | 1.40E-03 |
| TC + P | 7.02E-04 | 5.64E-04 |
| *C. perfringens* JGS1936 spores were treated with taurocholate (TC) and individual amino acids (represented by their respective one-letter code). Germination rates were calculated from the linear segment of optical density changes over time | | |
| GC + A | 1.85E-02 | 1.93E-03 |
| CH + A | 1.71E-03 | 4.54E-04 |
| TCD + A | 1.97E-02 | 2.11E-03 |
| CDC + A | 1.70E-03 | 8.81E-04 |
| TDC + A | 1.97E-02 | 2.15E-03 |
| DC + A | 2.25E-03 | 8.01E-04 |
| *C. perfringens* JGS1936 spores were treated with L-alanine (A) and either glycocholate (GC), cholate (Ch), taurochenodeoxycholate (TCD), chenodeoxycholate (CDC), taurodeoxycholate (TCD), or deoxycholate (DC). Germination rates were calculated from the linear segment of optical density changes over time | | |
