# Supplementary figures and images for: Comparison of sporulation and germination conditions for Clostridium perfringens type A and G strains
Source: Front Microbiol. 2023 May 9;14:1143399. doi: 10.3389/fmicb.2023.1143399 (PMC10203408; doi:10.3389/fmicb.2023.1143399)

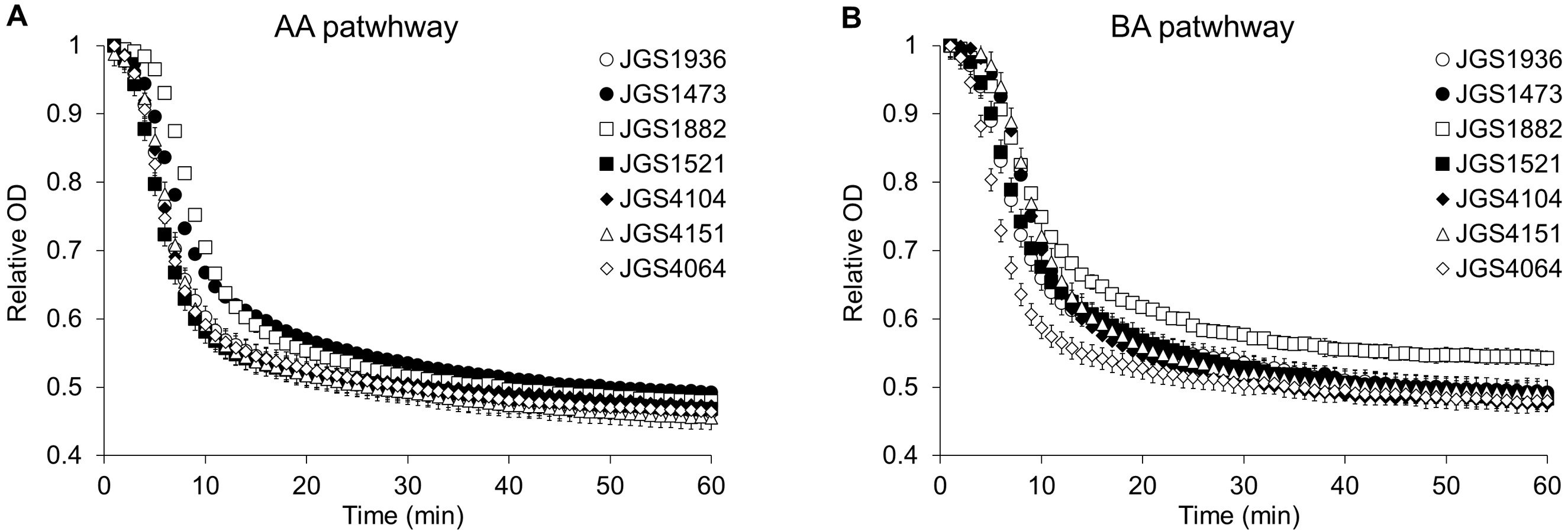

Supplement: Supplementary file 2 [file Image_1.JPEG]
